# Inequalities in complete pneumococcal vaccination among Peruvian children before and after the COVID-19 pandemic: An evaluation using demographic and health surveys from 2018 to 2023

**DOI:** 10.1017/S0950268826101332

**Published:** 2026-03-27

**Authors:** Brayan E. Gonzales, Theresa J. Ochoa, Marianne A. B. van der Sande

**Affiliations:** 1 Department of Public Health, Institute of Tropical Medicine, Antwerp, Belgium; 2 Instituto de Medicina Tropical Alexander von Humboldt, Universidad Peruana Cayetano Heredia, Lima, Peru; 3 Laboratory of Applied Microbiology and Biotechnology, Department of Bioscience Engineering, University of Antwerp, Antwerp, Belgium; 4 Facultad de Medicina, Universidad Peruana Cayetano Heredia, Lima, Peru; 5 Julius Global Health, University Medical Centre Utrecht, Utrecht, Netherlands

**Keywords:** COVID-19, inequalities, PCV13, Peru, vaccination coverage

## Abstract

This study assessed changes in complete pneumococcal vaccination coverage (CPVC) among Peruvian children <5 years before and after the COVID 19 pandemic and evaluated regional differences, associated sociodemographic factors and wealth-related inequality. 2018–2023 Demographic and Health Surveys (DHS) was analyzed. CPVC was defined as receiving the full 2 + 1 schedule of the 13 valent pneumococcal vaccine. Children aged 13–60 months were included. Multivariable analysis used modified Poisson regression and wealth related inequality was assessed using the Concentration index and Erreygers’s corrected Concentration index at national and regional levels. Among 95,586 children, CPVC decreased from 71.9% in 2019 to 69.4% in 2020 (*p* = 0.003), then returned to pre Covid levels from 2021 onward (72.2% in 2023; *p* = 0.001), particularly in Lima Metropolitana. Puno (53.3–58.6%) and Madre de Dios (50.9–62.1%) consistently showed the lowest coverage. Nationally, wealth- or sociodemographic related inequalities were minimal; however, regional interactions indicated that the effect of wealth on CPVC varied by area. Depending on the region, factors such as age group, household members and mather’s education were associated with lower CPVC, whereas age at first pregnancy, institutional birth, antenatal care and access to information increased CPVC. Ucayali showed persistently higher CPVC among wealthier populations. Despite a temporary decline during the pandemic, CPVC in Peru rapidly recovered, although regional gaps persist.

## Introduction

Immunization programmes are among the most cost-effective public health strategies to prevent targeted infectious diseases [1]. Immunization saves millions of lives annually by protecting against around 20 deadly diseases [2]. However, about 20 million children <1 year old remain unvaccinated each year; mainly in rural or conflict-affected areas with limited access to health services [2]. As a result, the World Health Organization (WHO) launched the ‘*Immunization Agenda 2030*’ (IA 2030), recognizing vaccination as a fundamental human right and a critical investment in global health and equity [2, 3]. IA 2030 promotes vaccine coverage as an indicator of health equity and calls for new policies to strengthen immunization programmes.

In 2015, invasive pneumococcal infection caused an estimated 300,000 deaths in children <5 worldwide [4]. Pneumococcus is considered a leading bacterial cause, identified in 10–25% of cases [5]. In 2018, Peru reported over 28,000 pneumonia cases in this age group, with a fatality rate of 1.04 per 100 episodes of pneumonia and a mortality rate of 10.5 per 100,000 [6]. To prevent invasive disease, Peru introduced the 7-valent pneumococcal conjugate vaccine (PCV7) in 2009, later replacing it with PCV10 in 2012 and PCV13 in 2015 [7].

Over the last decade, the WHO and Strategic Advisory Group of Experts on Immunization (SAGE) Global Vaccine Action Plan 2011–2020 highlighted how income level, socioeconomic status, and geography impact vaccination coverage. For instance, Diphteria-tetanus-pertusis (DTP3) and measles (MMR) vaccine coverage was 16% and 15% less in low-income countries than in high-income countries, respectively. In some countries, MMR coverage was also 33% lower in rural areas than in urban settings and 58% higher in the wealthiest quintile compared to the poorest [8]. Additional factors such as distance to health centres, maternal education and household income may contribute to low coverage, particularly in low- and middle-income countries. Similarly, poor urban communities, high migration areas, and regions with indigenous populations often exhibit persistently low vaccination coverage [8, 9]. Discrimination linked to migration may increase vaccine hesitancy and lower coverage [10].

The complex interplay of these variables may promote heterogeneous effects across geographic areas, even within the same country. An additional indicator of disparities in Peru’s health services is the density of human health resources. In 2016, the administrative regions of Callao and Lima reported the highest densities of physicians nationwide, with 25.0 and 19.4 per 10,000 population, respectively, whereas the administrative regions of Huánuco and Loreto region reported the lowest densities (5.3 and 5.7 per 10,000 population, respectively) [11].

According to Peruvian Ministry of Health (MoH) reports, complete PCV13 pneumococcal vaccination coverage (CPVC) declined already prior to the COVID-19 pandemic [12,13]. However, the pandemic’s impact on vaccination coverage, regional variations, and related inequalities in Peru remains undescribed. This study assessed CPVC coverage before, during, and after COVID-19 and explored associated factors, regional differences, and inequalities affecting coverage in children <5 years old.

## Methods

### Study design and population

This retrospective study analysed repeated cross-sectional data from the 2018 to 2023 Peruvian Demographic and Health Surveys (DHS).

The DHS is nationally and regionally representative survey, covering variables, such as area of residence (urban and rural), natural region, and administrative region. It provides annual data on demographics and child and maternal health, supporting the monitoring of Budget Program indicators, and informing health policy. The DHS is conducted yearly by the National Institute of Statistics and Informatics of Peru and includes (i) a household questionnaire; (ii) an individual questionnaire for females between 12 and 49 years old (including questions on the immunization status of their young children); and (iii) a health questionnaire for people who are ≥15 years old. Members of the household were defined as people who are currently resident in or spend the night at the household before the survey day [14]. This study included only households and mothers with children aged 13 to 60 months. Children without a vaccination card or presenting a card with incomplete information at the time of the survey were excluded.

DHS samples in each year were multistage, probabilistic, stratified, and using a sampling frame for each sampling stages performed by the National Institute of Statistics and Informatics of Peru (INEI) every year. The sampling frame was selected using statistical and cartographic information from the XII National Census of Populations and VII National Census of Households. This sample design was included from DHS-2015 and improved the statistical precision of the indicators and decreased the coefficients of variation at less than 15% [15].

### Variables

Peru has a 2 + 1 dose PCV schedule: the first dose at the age of 2 months, the second dose at the age of 4 months, and the last dose (booster) at the age of 12 months [16,17]. CPVC was defined as having completed the vaccination schedule of the PCV13 (as documented on the vaccination card) at the time of survey using the variable *S45NM3* from each DHS. Wealth index was a composite measure of a household’s cumulative living standard. It was calculated using information on household ownership of selected assets (e.g., televisions, bicycles), materials used for housing construction, and the type of water access and sanitation facilities available [18]. Wealth index and the breakpoints of quintiles were determined by year according to the wealth distribution of each DHS. Other covariates are described with an operational definition in Supplementary Table S1.

### Descriptive analysis

The six databases corresponding to the DHS surveys carried out between 2018 and 2023 were merged in a new database for the analysis. The demographic and epidemiological data were described with absolute and relative frequencies or means (with standard deviation). As the DHS uses a complex, multistage sampling design, all proportion estimates were calculated using survey weights to account for unequal probabilities of selection and to obtain nationally representative results (stratification and clustering variables were also incorporated to ensure valid variance estimation). Specifically, all proportions were weighted using the expansion factor (Variable: ‘*V005*’ or ‘*Factor mujer*’), stratum (Variable: ‘H*V022*’ or ‘*estrato*’) and cluster (Variable: ‘*HV001*’ or ‘*Conglomerado*’), according to the recommendation of Peruvian DHS [14] Additionally, a map of Peru was developed to show the proportion of children <5 years old with complete PCV13 vaccination per year and region.

### Bivariate and multivariable analysis

Statistical analysis was performed using χ^2^ test and adjusted Wald test for population characteristics and CPVC. Bivariate and multivariable analysis employed modified Poisson regression with linearized variance to estimate prevalence ratios (PR) and adjusted prevalence ratios (aPR). Given the heterogeneity across Peruvian natural regions (Lima Metropolitana, Coast, Highland and Jungle), stratified analysis by natural region were conducted. To test whether the effect of wealth on CPVC varied by natural region, interaction terms (‘*##*’ operator) were included. Adjusted predicted probabilities (marginal effects) of CPVC across wealth strata and regions were plotted (Supplementary Figure S2). All analyses were carried out using complex survey package (‘*svy*’) in Stata/SE v.19.5 program (Stata Corporation, TX, USA). Due to the multiple comparisons, significance was set at *p* < 0.001 with 95% confidence intervals (95% CI).

### Inequality analysis

The wealth index was used to assess to what extent CPVC was associated with wealth. The Concentration index (Ci) [19] and Erreygers’s corrected Concentration index were estimated using the wealth index (as continuous variable) to assess CPVC-specific socioeconomic populations [20–22] in national level and per administrative region (the 25 administrative regions of Peru).

### Ethical aspects

The Peruvian DHS is an anonymized open access dataset (https://proyectos.inei.gob.pe/endes/) that does not include any personally identifiable information. Therefore, ethical approval was not required for its use.

## Results

### Population’s characteristics

Among 95,926 children aged 13–60 months across the six evaluated DHS, 0.36% were excluded for lacking a valid vaccination card at the time of the survey, resulting in a total of 95,586 Peruvian children included in this analysis; 50.9% were males. The median age was 36.4 months (±13.5) and 22.3% were between 13 and <24 months. Overall, 27.2% lived in Lima Metropolitana and 74.0% in urban areas (including Lima) (Table 1 and Supplementary Table S2). Regarding maternal characteristics, 21.0% had their first delivery before 18 years old, 19.0% had not completed primary education, 24.9% belonged to the poorest wealth quintile, and 6.1% had delivery at home. In addition, 64.3% lacked access to social programmes, and 9.1% had no access to information (access to TV, radio, or internet). CPVC during these years averaged 71.1% (Table 1 and Supplementary Table S3).

**Table 1.** Demographic and sociodemographic characteristics associated with complete pneumococcal vaccination coverage in Peruvian children <5 years old between 2018 and 2023 (N = 95,586)^a^

Characteristics	*N* = 95,586 n (%)	Complete pneumococcal vaccinated		*p* ^b^
		No	Yes	
		*n* = 28,175	*n* = 67,411	
		*n* (%)	*n* (%)	
Sex				0.815
Male	48,696 (50.9)	14,269 (28.9)	34,427 (71.1)	
Female	46,890 (49.1)	13,906 (28.8)	32,984 (71.2)	
Age (months)^c^	36.4 ± 13.5	37.4 ± 13.8	35.9 ± 13.4	<0.001^d^
Age group				<0.001
13– <24	21,505 (22.3)	5,955 (27.5)	15,550 (72.5)	
24– <36	24,029 (25.2)	6,341 (25.9)	17,688 (74.2)	
36– <48	24,914 (26.1)	7,430 (29.4)	17,484 (70.6)	
48– <60	25,138 (26.4)	8,449 (32.4)	16,689 (67.7)	
Natural region of residence				<0.001
Lima metropolitan^a^	11,931 (27.2)	3,704 (30.9)	8,227 (69.1)	
Coast	28,471 (27.3)	8,295 (27.9)	20,176 (72.1)	
Highlands	31,274 (27.5)	8,745 (27.7)	22,529 (72.3)	
Jungle	23,910 (18.0)	7,431 (29.0)	16,479 (71.0)	
Area of residence				<0.001
Urban	66,832 (74.0)	19,850 (29.4)	46,982 (70.6)	
Rural	28,754 (26.0)	8,325 (27.3)	20,429 (72.7)	
Family size (members)				<0.001
Small (<4)	18,087 (18.2)	4,510 (23.9)	13,577 (76.1)	
Medium (4–5)	61,030 (63.3)	17,580 (28.2)	43,450 (71.8)	
Large (≥6)	16,469 (18.5)	6,085 (36.1)	10,384 (63.9)	
Mother’s age at first delivery				<0.001
<18	21,815 (21.0)	7,692 (35.1)	14,123 (64.9)	
18–30	68,155 (72.1)	19,218 (27.7)	48,937 (72.3)	
>30	5,616 (6.9)	1,265 (22.4)	4,351 (77.6)	
Mother’s education				<0.001
Primary/no education	18,986 (19.0)	5,765 (30.0)	13,221 (70.1)	
Secondary	45,093 (46.2)	13,713 (29.7)	31,380 (70.3)	
Higher	31,507 (34.8)	8,697 (27.2)	22,810 (72.8)	
Wealth index^b^	0.23 ± 0.99	0.22 ± 1.0	0.25 ± 1.0	0.024^d^
Wealth index (quintile)				0.671
Most poor	27,644 (24.9)	8,419 (29.2)	19,225 (70.8)	
Poor	25,278 (23.8)	7,482 (28.8)	17,796 (71.2)	
Middle	19,001 (20.2)	5,491 (29.1)	13,510 (70.9)	
Rich	14,077 (17.2)	4,067 (28.2)	10,010 (71.8)	
Most rich	9,586 (13.9)	2,716 (28.7)	6,870 (71.3)	
Place of delivery				<0.001
At home	5,504 (6.1)	2,277 (38.0)	3,227 (62.0)	
Health facility	88,753 (94.0)	25,198 (27.7)	63,555 (72.3)	
Antenatal care (visits)				<0.001
0–6	14,718 (18.3)	5,384 (35.8)	9,334 (64.2)	
7–12	57,833 (70.6)	15,806 (26.9)	42,027 (73.1)	
≥13	8,278 (11.1)	2,098 (24.7)	6,180 (75.3)	
Access to social programmes				<0.001
No	58,183 (64.3)	19,175 (32.0)	39,008 (68.0)	
Yes	37,403 (35.7)	9,000 (23.3)	28,403 (76.7)	
Information access^e^				<0.001
No	10,160 (9.1)	3,631 (34.9)	6,529 (65.2)	
Yes	85,426 (90.9)	24,544 (28.3)	60,882 (71.7)	
DHS period				<0.001
2018	18,372 (19.2)	5,583 (29.5)	12,789 (70.5)	
2019	16,555 (17.3)	4,621 (28.1)	11,934 (71.9)	
2020	10,445 (10.9)	3,218 (30.6)	7,227 (69.4)	
2021	17,256 (18.1)	5,212 (29.4)	12,044 (70.6)	
2022	16,820 (17.6)	4,942 (27.9)	11,878 (72.1)	
2023	16,138 (16.9)	4,599 (27.8)	11,539 (72.2)	

All proportions were weighted.

a Some variables may add less than 95,586 due to missing data.

b Mean ± standard deviation.

c χ^2^ adjusted for complex survey design.

d Adjusted Wald test.

e Access to internet, TV, and radio.

### Complete PCV13 vaccination coverage

**Figure 1.**
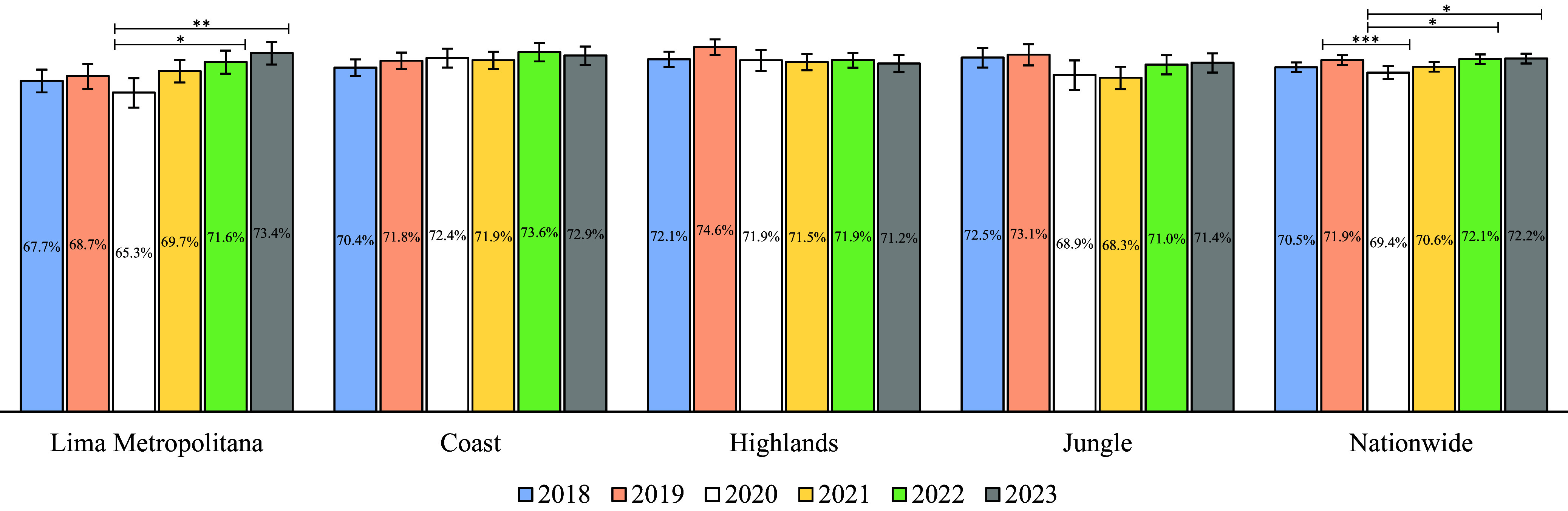
National estimation of complete pneumococcal vaccination coverage in Peruvian children <5 years old between 2018 and 2023. *p = 0.001, **p < 0.001, ***p = 0.003.

At the national level, CPVC in 2020 (69.4%) showed a 2.5% decrease compared to the previous pre-COVID year (72.2%; *p* = 0.003). This was followed by a recovery in coverage over the subsequent years, reaching 72.2% in 2023 (*p* = 0.001) (Figure 1). At the natural regional level, a similar decline in coverage was observed in 2020 across all natural regions, followed by a recovery in the following years except for the Highlands, where coverage levels did not return to pre-COVID levels. These trends were not statistically significant in most natural regions, except for Lima Metropolitana, where coverage from 2020 (65.3%) increased significantly in 2022 (71.6%) (*p* = 0.001) and 2023 (73.4%) (*p* < 0.001) (Figure 1).

When the administrative regional coverage was evaluated in more detail (Supplementary Figure S1), Puno (53.3–58.6%) (in the Highlands), and Madre de Dios (50.9–52.1%) (in the Jungle) constantly reported the lowest coverage levels over the 6 years. Administrative regions in northern Peru generally showed the highest coverage levels, particularly Huánuco (83.7%) in 2018 and (83%) 2019; San Martín (78.9%) in 2020; Cajamarca (78%) in 2021; La Libertad (81.2%) in 2022; and Ancash (79.1%) in 2023 (Supplementary Table S4).

### Factors associated with complete PCV13 vaccination

Nationwide, CPCV was higher among children living in rural areas, with a smaller family size, higher maternal age, born in health facility, increased antenatal care, and access to social programme and information (*p* < 0.001) (Supplementary Table S5).

In the multivariable model, CPCV was higher in the Coast (aPR:1.04; *p* < 0.001) and the Jungle (aPR 1.06; *p* < 0.001) compared to Lima Metropolitana. Interaction was observed between the effect of wealth index and natural region, showing a positive effect of wealth on CPCV in the Highlands (Supplementary Table S5), but not in other regions, except in two administrative regions (Amazonas and Ucayali) in the Jungle region (Supplementary Figure S2).

Stratified analysis by natural regions showed that older age in the Coast (aPR:0.91) and Highlands (aPR:0.92); living in a household with 4–5 members in all natural regions (aPR:0.92–0.96) except in the Jungle; and having mothers with only secondary education (aPR:0.94) in the Highlands, were significantly (*p* < 0.001) associated with a decreased prevalence of being fully vaccinated (Table 2). In contrast, in all natural regions except in the Highlands having a mother whose first pregnancy occurred at 18–30 years old (aPR:1.05–1.18); in the Jungle a delivery at health facility (aPR:1.17); and in all natural regions except in Lima Metropolitana having a mother with many (7–12) antenatal care visits (aPR:1.08–1.17) and information access (aPR:1.07–1.10) were significantly associated (*p* < 0.001) with an increased prevalence of being fully vaccinated. Additionally, only in the Jungle, being from the most poor wealth quintile (aPR:0.92; 95% CI:0.88–0.95) or from the second poorest wealth quintile (aPR:0.93; 95% CI: 0.90–0.96) was associated with a lower prevalence of being fully vaccinated (*p* < 0.001) (Table 2).

**Table 2.** Factors associated with complete pneumococcal vaccination coverage stratified by natural region (multivariate analysis)^a^

Characteristics		Lima metropolitan^a^			Coast			Highlands			Jungle		
		aPR	95% CI	*p*	aPR	95% CI	*p*	aPR	95% CI	*p*	aPR	95% CI	*p*
Sex													
	Male	Ref.			Ref.			Ref.			Ref.		
	Female	1.02	0.99–1.05	0.210	1.00	0.98–1.02	0.861	1.01	0.99–1.03	0.219	0.98	0.96–1.00	0.085
Age group (months)													
	13– <24	Ref.			Ref.			Ref.			Ref.		
	24– <36	1.01	0.97–1.05	0.652	1.02	1.00–1.05	0.075	1.01	0.99–1.03	0.516	1.03	1.00–1.06	0.029
	36– <48	0.97	0.93–1.01	0.197	0.96	0.94–0.98	0.001	0.96	0.94–0.99	0.002	1.00	0.97–1.03	0.947
	48– <60	0.93	0.89–0.97	0.002	0.91	0.89–0.94	<0.001	0.92	0.89–0.94	<0.001	0.97	0.94–1.00	0.028
Type of residence													
	Urban	–	–	–	Ref.			Ref.			Ref.		
	Rural	–	–	–	1.03	0.99–1.07	0.148	1.03	1.00–1.06	0.036	1.00	0.97–1.03	0.887
Family size (members)													
	Small (<4)	Ref.			Ref.			Ref.			Ref.		
	Medium (4–5)	0.92	0.89–0.96	<0.001	0.96	0.93–0.98	<0.001	0.94	0.92–0.96	<0.001	0.97	0.95–0.99	0.012
	Large (≥6)	0.85	0.81–0.89	<0.001	0.87	0.84–0.89	<0.001	0.86	0.83–0.89	<0.001	0.87	0.84–0.90	<0.001
Mother’s age at first delivery													
	<18	Ref.			Ref.			Ref.			Ref.		
	18–30	1.18	1.12–1.25	<0.001	1.10	1.07–1.13	<0.001	1.03	1.00–1.05	0.019	1.05	1.03–1.08	<0.001
	>30	1.25	1.17–1.33	<0.001	1.17	1.13–1.22	<0.001	1.09	1.05–1.14	<0.001	1.10	1.04–1.16	<0.001
Mother’s education													
	Primary/no education	Ref.			Ref.			Ref.			Ref.		
	Secondary	1.04	0.97–1.12	0.284	1.00	0.97–1.03	0.937	0.95	0.92–0.97	<0.001	1.01	0.99–1.04	0.337
	Higher	1.06	0.98–1.14	0.159	1.01	0.98–1.05	0.458	0.96	0.93–0.99	0.006	1.00	0.97–1.04	0.787
Wealth index (quintile)													
	Most poor	0.84	0.72–0.98	0.028	0.96	0.91–1.00	0.049	0.98	0.94–1.01	0.220	0.92	0.88–0.95	<0.001
	Poor	0.98	0.94–1.03	0.523	0.99	0.97–1.01	0.413	0.98	0.95–1.01	0.265	0.93	0.90–0.96	<0.001
	Middle	Ref.			Ref.			Ref.			Ref.		
	Rich	1.02	0.98–1.06	0.384	0.99	0.97–1.02	0.639	1.05	1.01–1.09	0.019	0.97	0.93–1.00	0.089
	Most rich	1.00	0.95–1.04	0.898	0.98	0.95–1.01	0.229	1.06	1.01–1.11	0.015	1.00	0.95–1.06	0.875
Place of delivery													
	At home	Ref.			Ref.			Ref.			Ref.		
	Health facility	0.93	0.72–1.19	0.553	1.06	0.95–1.19	0.261	1.07	1.03–1.12	0.001	1.17	1.11–1.24	<0.001
Antenatal care (visits)													
	0–6	Ref.			Ref.			Ref.			Ref.		
	7–12	1.08	1.03–1.12	<0.001	1.12	1.09–1.15	<0.001	1.11	1.08–1.14	<0.001	1.17	1.13–1.21	<0.001
	≥13	1.10	1.04–1.16	0.001	1.14	1.10–1.19	<0.001	1.16	1.12–1.20	<0.001	1.21	1.16–1.26	<0.001
Access to social programmes													
	No	Ref.			Ref.			Ref.			Ref.		
	Yes	1.07	1.03–1.10	<0.001	1.12	1.10–1.15	<0.001	1.21	1.19–1.24	<0.001	1.20	1.17–1.23	<0.001
Information access													
	No	Ref.			Ref.			Ref.			Ref.		
	Yes	1.03	0.92–1.15	0.599	1.10	1.05–1.16	<0.001	1.07	1.04–1.10	<0.001	1.09	1.06–1.13	<0.001
DHS period													
	2018	1.03	0.97–1.10	0.316	0.95	0.92–0.99	0.014	0.98	0.94–1.02	0.254	1.03	0.98–1.08	0.227
	2019	1.07	1.01–1.13	0.028	0.99	0.96–1.03	0.599	1.03	0.99–1.06	0.165	1.05	1.00–1.10	0.057
	2020	Ref.			Ref.			Ref.			Ref.		
	2021	1.08	1.02–1.14	0.013	0.98	0.94–1.02	0.255	0.98	0.95–1.02	0.322	0.97	0.92–1.02	0.275
	2022	1.11	1.05–1.17	0.001	1.01	0.98–1.05	0.471	0.98	0.94–1.02	0.316	1.01	0.97–1.06	0.601
	2023	1.11	1.05–1.18	<0.001	1.00	0.96–1.04	0.870	0.98	0.94–1.01	0.201	1.01	0.97–1.06	0.602

aPR, Adjusted Prevalence ratio; 95% CI, 95% confidence interval; Ref, Reference category.

a Multivariate analysis using linearized variance (modified) Poisson regression. Adjusted by sex, age group, type of residence, family size, mother’s age at first delivery, mother’s education, wealth index, place of delivery, antenatal care, access to social programmes and information access, and DHS period.

### Inequality evaluation

At the national level, CPVC was higher among populations with a higher wealth index in 2022 [Ci: 0.012 (*p* < 0.001); ECi: 0.035 (*p* < 0.001)] and 2023 [Ci: 0.016 (*p* < 0.001); ECi: 0.048 (*p* < 0.001)] (Table 3 and Supplementary Table S6).

**Table 3.** Concentration index for complete pneumococcal vaccination coverage according to wealth index (continuous) between 2018 and 2023

AdministrativeRegion	Concentration index																	
	DHS-2018			DHS-2019			DHS-2020			DHS-2021			DHS-2022			DHS-2023		
	Ci	CI 95%	*p*	Ci	CI 95%	*p*	Ci	CI 95%	*p*	Ci	CI 95%	*p*	Ci	CI 95%	*p*	Ci	CI 95%	*p*
Nationwide	−0.009	−0.018 to −0.001	0.036	−0.003	−0.011 to 0.006	0.574	−0.006	−0.017 to 0.006	0.341	0.014	0.005 to 0.023	0.002	0.012	0.004 to 0.020	<0.001	0.016	0.008 to 0.025	<0.001
Amazonas	0.080	0.038 to 0.122	<0.001	0.055	0.017 to 0.094	0.005	0.077	0.012–0.143	0.022	0.066	0.021 to 0.112	0.005	0.125	0.071 to 0.180	<0.001	0.172	0.122 to 0.221	<0.001
Ancash	−0.012	−0.038 to 0.014	0.378	0.005	−0.020 to 0.029	0.694	−0.036	−0.087 to 0.015	0.164	−0.018	−0.056 to 0.020	0.343	−0.030	−0.063 to 0.004	0.079	−0.030	−0.058 to –0.003	0.030
Apurimac	0.012	−0.020 to 0.045	0.462	0.012	−0.022 to 0.046	0.481	−0.008	−0.044 to 0.028	0.660	0.014	−0.019 to 0.048	0.401	0.003	−0.026 to 0.032	0.846	0.000	−0.030 to 0.031	0.985
Arequipa	0.027	−0.006 to 0.059	0.110	0.034	0.004 to 0.063	0.025	0.041	−0.018 to 0.099	0.170	0.025	−0.015 to 0.065	0.220	0.023	−0.018 to 0.063	0.276	0.008	−0.018 to 0.034	0.566
Ayacucho	0.015	−0.018 to 0.049	0.363	−0.037	−0.067 to −0.008	0.015	0.023	−0.011 to 0.056	0.182	0.015	−0.014 to 0.043	0.304	0.004	−0.023 to 0.032	0.757	0.015	−0.017 to 0.047	0.345
Cajamarca	0.020	−0.009 to 0.048	0.181	0.011	−0.021 to 0.042	0.505	0.028	−0.021 to 0.077	0.253	0.011	−0.019 to 0.041	0.464	−0.009	−0.040 to 0.021	0.545	0.039	0.004–0.074	0.029
Callao	0.015	−0.018 to 0.047	0.363	−0.006	−0.040 to 0.028	0.735	0.060	0.012 to 0.107	0.015	0.010	−0.026 to 0.046	0.587	0.035	0.002–0.068	0.037	0.009	−0.019 to 0.037	0.516
Cusco	−0.012	−0.040 to 0.015	0.373	−0.015	−0.043 to 0.012	0.273	−0.018	−0.067 to 0.031	0.459	−0.003	−0.030 to 0.025	0.846	0.008	−0.022 to 0.039	0.591	−0.014	−0.050 to 0.022	0.442
Huancavelica	−0.010	−0.043 to 0.023	0.565	−0.004	−0.040 to 0.033	0.851	0.035	−0.006 to 0.076	0.093	0.006	−0.024 to 0.036	0.681	0.018	−0.014 to 0.050	0.274	0.013	−0.023 to 0.048	0.482
Huánuco	−0.001	−0.021 to 0.020	0.946	−0.010	−0.028 to 0.009	0.308	0.024	−0.019 to 0.067	0.266	0.008	−0.021 to 0.037	0.605	0.012	−0.020 to 0.043	0.465	0.009	−0.018 to 0.036	0.532
Ica	0.026	−0.020 to 0.072	0.261	0.076	0.045–0.108	<0.001	−0.004	−0.048 to 0.041	0.864	−0.002	−0.033 to 0.030	0.923	0.045	0.001–0.089	0.046	0.009	−0.030 to 0.048	0.654
Junín	0.014	−0.024 to 0.052	0.463	0.027	−0.014 to 0.067	0.194	0.023	−0.021 to 0.066	0.311	−0.006	−0.035 to 0.024	0.700	0.005	−0.022 to 0.031	0.736	0.017	−0.010 to 0.044	0.222
La Libertad	−0.006	−0.044 to 0.032	0.759	0.000	−0.031 to 0.032	0.997	0.015	−0.019 to 0.050	0.378	0.041	0.006 to 0.077	0.024	0.009	−0.024 to 0.041	0.600	0.012	−0.026 to 0.050	0.532
Lambayeque	0.026	−0.022 to 0.074	0.281	0.020	−0.020 to 0.058	0.325	0.018	−0.025 to 0.060	0.407	−0.010	−0.050 to 0.030	0.621	0.011	−0.033 to 0.055	0.620	0.035	−0.025 to 0.096	0.253
Lima	0.015	−0.005 to 0.034	0.147	0.019	−0.003 to 0.040	0.089	−0.009	−0.032 to 0.015	0.475	0.029	0.009 to 0.049	0.005	0.030	0.013–0.046	<0.001	0.011	−0.007 to 0.029	0.224
Loreto	0.010	−0.026 to 0.045	<0.001	0.028	−0.014 to 0.069	0.192	0.046	−0.024 to 0.117	0.194	0.090	0.034 to 0.146	0.002	0.084	0.039–0.130	<0.001	0.095	0.053–0.137	<0.001
Madre de Dios	0.044	0.001–0.087	0.048	0.005	−0.033 to 0.043	0.795	0.043	−0.032 to 0.117	0.256	0.027	−0.022 to 0.077	0.279	0.037	−0.021 to 0.094	0.211	0.063	0.013–0.114	0.015
Moquegua	0.012	−0.028 to 0.052	0.544	−0.014	−0.045 to 0.017	0.379	0.003	−0.041 to 0.047	0.901	0.032	−0.002 to 0.067	0.062	0.014	−0.030 to 0.057	0.535	−0.013	−0.045 to 0.019	0.427
Pasco	−0.023	−0.068 to 0.022	0.308	−0.010	−0.044 to 0.025	0.568	0.011	−0.045 to 0.067	0.700	0.010	−0.025 to 0.044	0.575	−0.006	−0.052 to 0.040	0.800	0.006	−0.033 to 0.044	0.775
Piura	−0.013	−0.039 to 0.014	0.346	−0.039	−0.069 to –0.008	0.013	−0.016	−0.048 to 0.017	0.340	0.019	−0.008 to 0.047	0.170	−0.020	−0.055 to 0.014	0.241	−0.010	−0.041 to 0.022	0.544
Puno	−0.083	−0.136 to −0.030	0.002	−0.040	−0.097 to 0.018	0.178	−0.085	−0.153 to –0.017	0.015	−0.048	−0.096 to 0.000	0.048	−0.020	−0.075 to 0.034	0.458	0.000	−0.054 to 0.055	0.989
San Martin	0.007	−0.025 to 0.039	0.675	0.025	−0.006 to 0.056	0.110	0.018	−0.012 to 0.048	0.241	0.025	−0.010 to 0.059	0.161	0.047	0.010–0.084	0.013	0.021	−0.009 to 0.052	0.167
Tacna	0.015	−0.039 to 0.068	0.587	0.009	−0.028 to 0.046	0.641	0.025	−0.036 to 0.085	0.420	0.032	0.001 to 0.063	0.042	0.037	0.004–0.070	0.027	−0.012	−0.042 to 0.018	0.437
Tumbes	0.010	−0.019 to 0.038	0.499	0.037	0.006 to 0.068	0.020	−0.008	−0.048 to 0.033	0.714	−0.001	−0.029 to 0.027	0.943	0.006	−0.019 to 0.030	0.655	0.001	−0.026 to 0.028	0.963
Ucayali	0.095	0.060 to 0.130	<0.001	0.073	0.038 to 0.109	<0.001	0.089	0.044 to 0.134	<0.001	0.149	0.101 to 0.197	<0.001	0.113	0.070–0.156	<0.001	0.091	0.055–0.126	<0.001

Ci, Concentration index.

*p* < 0.05: Index probability is different from zero (inequality).

When inequities in the CPVC by administrative region were evaluated, in 2018, coverage was higher among populations with a lower wealth index in Puno (in the Highlands) and among those with a higher wealth index in Ucayali (in the Jungle) (Table 3 and Supplementary Table S6). In 2019, coverage was higher among populations with a higher wealth index in Ica and Ucayali (in the Coast and the Jungle, respectively) (Table 3 and Supplementary Table S6). Between 2020 and 2023, vaccination coverage in Ucayali remained higher among populations with a higher wealth index (Table 3 and Supplementary Table S6).

## Discussion

Changes in CPVC in Peru related to the COVID-19 pandemic in complete PCV13 immunization were analysed in this study. The probability of children being fully PCV13 immunized was lower in 2020 compared to 2022 and 2023. Sociodemographic characteristics and economic inequality (determined by wealth quintiles) were not independently associated with vaccination coverage at the national level but differed by region. We also observed interaction between wealth and regions. In only one administrative region (Ucayali in Jungle region), CPVC was concentrated among the population with a higher wealth index, a pattern not observed in other administrative regions. The observed subregional variations imply that such detailed analysis is needed to identify populations in need of vaccination support, which might be masked by national or regional level analysis.

These findings in Peru mirror the broader Latin American and Caribbean context, where routine childhood immunization faced major COVID‑19 disruptions that exceeded expect decline [23]. Early in the COVID‑19 pandemic, international agencies warned that restrictions and reduced service access could disrupt routine vaccination and cause missed doses [24]. Evidence from Latin America shows variable pandemic‑period changes in PCV coverage, with studies from Argentina, Brazil, Chile, Colombia, and Mexico reporting declines of different magnitudes in vaccination coverage [25]. Regional analyses suggest that the pandemic worsened inequities, with socioeconomic indicators linked to greater coverage declines [26], which aligns with the wealth and region-based gaps reported in this study. Differences in decline and recovery patterns between Peru and other countries may reflect varying health system strain and catch‑up strategies.

According to a report of MoH of Peru, national PCV13 coverage (three doses) was 93.7%, 80.3%, 73.6%, 75.2%, 70.7%, and 79.9% between 2018 and 2023, respectively [12, 13]. These values were 4.2–23.2% higher than observed in this study. MoH data also showed a 6.7% decrease in 2020 (compared to 2019) and this reduction persisted until 2023, whereas our results indicated post-2020 recovery. Coverage estimates of the MoH are based on the ‘HISMINSA’ system, which records daily doses and vaccine types administered nationwide. Data are cross-referenced with the ‘District nominal register of children under six years old’ (DNRC) [17], continuously updated by healthcare workers in collaboration with the ‘National Registry of Identification and Civil Status’ (RENIEC). However, the MoH coverage calculation may be affected by denominator error if DNRC does not fully capture the true eligible population. Incomplete or delayed registration (e.g., delays in RENIEC enrolment), internal migration and limited access to health and civil registration services, particularly in rural areas with frequent home deliveries [27, 28] can lead to under-enumeration of children in DNRC. An undercounted DNRC denominator would inflate coverage estimates, even when the number of administered doses is accurate, resulting in apparent overestimation. The lower coverage reported in this study using DHS data compared with MoH reports, likely reflects these limitations, particularly during the first 2 years of the COVID-19 pandemic.

In 2020, CPVC declined nationwide by 2.5%, aligning with the beginning of the COVID-19 pandemic. Although in this study, causality cannot be confirmed, several factors likely contributed: (i) lockdown restrictions limiting access to non-COVID-19 services; (ii) reallocation of health workers to pandemic care [29–31]; (iii) shortage of well-child visits, outpatient consultation and home immunization programmes; (iv) frequent changes in the MoH, with three ministers in the first 4 months [32]; and (v) ‘Vacunagate’ scandal, in which politicians and health leaders, including the President of Peru, received experimental vaccines, generating mistrust and which led to his resignation [33–36]. These issues contributed to hesitancy among the population to accept COVID-19 immunization, which may have extended to routine immunization [37, 38]. Methodological changes in DHS may also explain some of the differences. DHS-2020 developed initially tele-interviews and gradually returned to field interviews, while the other DHS were conducted entirely through field interviews. Those differences may have affected vaccination data collection, contributing to the observed decline in 2020. Further analysis of longer-term trends is needed to distinguish pandemic-related effects from normal fluctuations in coverage.

Factors at the level of natural regions and administrative regions associated with vaccination coverage were identified but without substantial changes in the measures of association. This contrasts with our hypothesis that coverage might differ between regions. Lima Metropolitana, the capital and largest Peruvian city, generally has better access to healthcare facilities than rural areas in the Highlands and Jungle. In these regions, barriers such as limited access to healthcare services [39], poor infrastructure and transportation [40, 41], lower socioeconomic status [22, 42], and reduced education or awareness of vaccination programmes [20, 22] may hinder access to immunization, including pneumococcal vaccines. Cultural practices, ethnicities, and religion can also influence healthcare-seeking behaviour and vaccination hesitancy [43–45]. It is encouraging that this analysis showed that overall coverage was comparable and stable, suggesting a strong nation-wide vaccination system. Addressing (sub)regional factors is needed to design targeted interventions to improve coverage and reduce socioeconomic inequalities where they still impact coverage.

Nationwide, our analysis showed a slightly higher coverage among wealthier populations, but this effect was minimal and not reflected in stratified geographical analysis, suggesting that economic inequalities had limited impact on CPVC. However, when stratifying by administrative region, we observed heterogeneity; whereby in Ucayali, CPVC was consistently higher among the wealthiest populations. This suggests that local factors, such as access to healthcare, infrastructure, or cultural practices can influence vaccination uptake even when no national trend is observed. Previous studies support these findings but only at national level. For example, DHS-2017 data found that measles immunization was not associated with wealth [46]. Similarly, DHS 2010–2019 data found no effect of wealth on complete diphtheria vaccination [41] and DHS-2021 indicated that wealth did not influence vaccine coverage of Bacille Calmette Guerin vaccine, combined diphtheria/tetanus and pertussis vaccine, polio vaccine, and measles-containing-vaccine first-dose during the COVID-19 pandemic [20]. Our study now adds to this as the first study in Peru to evaluate this for PCV13 uptake, confirming that, even pandemic-related disruption, economic inequalities did not affect coverage at the national level, although local variations exist.

Vaccination coverage differed by place of residence, with 70.6% in urban areas and 72.7% in rural areas (*p* = 0.001), contrary to expectations [8]. After stratification by natural region, this pattern persisted only in the Highlands. This heterogeneity may reflect uneven effects of vaccination-coverage interventions implemented by the Peruvian MoH across regions, consistent with the 2020 WHO/SAGE review highlighting that progress has not always translated into clearly measurable targets [8]. A methodological explanation is also plausible; the DHS classifies residents of Lima Metropolitana as ‘urban’ and the better access to healthcare services in the capital may inflate urban coverage overall; this effect may attenuate when analyses are stratified by region (specialty in the Coast and Jungle).

We assessed how factors associated with vaccination coverage in Peru evolved over time and how these may influence annual changes in coverage. A study from Mozambique (1997–2015) illustrated how these dynamics can shift, as between 1997 and 2003, vaccination coverage increased more among the poorest (14.4%) than the wealthier (2.1%) while in 2003–2011, coverage increased by 2.3% among the poorest but declined by 2.2% among the wealthier population. Afterwards the trend reversed, with higher coverage increases among the wealthier (2.8%) compared to the poorest (0.27%) population [47]. Such findings underscore the importance of understanding how demographic, social, and economic factors shape vaccination trends in relation to the Sustainable Development Goals of the Immunization Agenda 2030 [2].

The DHS data show similar trends in CPVC to those reported by the Ministry of Health of Peru, despite differences in the exact estimated rates before the COVID-19 disruption. Nevertheless, real-time vaccination data collection remains an area for improvement. Passive surveillance systems may be suboptimal in the Peruvian context, where certain populations lack adequate access to healthcare services and are not always registered in a timely manner in the ‘District nominal register of children under six years old’. Peru has made steady progress towards achieving universal vaccination coverage, which is reflected in the absence of independent factors that substantially modified CPVC during the evaluated years. However, targeted strategies are recommended to improve CPVC in populations and (sub)regions with persistently low coverage.

## Supporting information

10.1017/S0950268826101332.sm001 Gonzales et al. supplementary material Gonzales et al. supplementary material

## Data Availability

The raw data supporting the conclusions of this article will be made available by the authors, without undue reservation.

## Supplementary material

The supplementary material for this article can be found at http://doi.org/10.1017/S0950268826101332.
