# Supplementary material for: Inequalities in complete pneumococcal vaccination among Peruvian children before and after the COVID-19 pandemic: An evaluation using demographic and health surveys from 2018 to 2023
Source: Epidemiol Infect. 2026 Mar 27;154:e49. doi: 10.1017/S0950268826101332 (PMC13100934; doi:10.1017/S0950268826101332)
Supplement: Gonzales et al. supplementary material [file S0950268826101332sup001.pdf]

Table S1. Operational definition of variable

Variable	Definition	DHS variable code	Questionary resource
Sex	Child's biological sex	B4	REC21
Age	Months of age	HW1	RECH44
Natural region of residence	Natural region of residence (Lima Metropolitana, the Coast, the Highlands, the Jungle)	SREGION	REC91
Area of residence	Type of child's residence (urban, rural)	HV025	RECH0
Administrative region	Administrative region of residence (the 25 administrative regions)	HV023	RECH0
Family size	Number of family members that live with the child	HV009	RECH0
Mother's age at first delivery	Mother had her first delivery at <18, 18 - 24, ≥25	V212	RE223132
Mother´s education	Mother's educational level	V106	REC0111
Wealth index	Wealth index (continuous variable)	V191	REC0111
Wealth index (quintile)	Wealth index quintiles stratified	V190	REC0111
Place of delivery	Child's place of birth	M15	REC41
Antenatal care (visit)	Number of visits of antenatal care	M14	REC41
Access to social programs	Family has access to at least one social programs: BECA 18, TRABAJA PERU, JUNTOS, Cuna Más, PENSIÓN 65, Vaso de leche, Comedor popular, Cuna mas	QH91/93/95/99 /101/103/106	Programas Sociales x Hogar
Information access	Family access to mass media (internet, TV, or radio)	SH61Q/HV207 /HV208	REC23
DHS period	Year of the demographic and health survey (DHS)	ID1	DHS

Table S2. Demographic and sociodemographic characteristics of Peruvian children <5 years old between 2018 and 2023 (N=95,586) [a]

Characteristics	N=95,586 n (%)	DHS					
		2018 n=18,372 n (%)	2019 n=16,555 n (%)	2020 n=10,445 n (%)	2021 n=17,256 n (%)	2022 n=16,820 n (%)	2023 n=16,138 n (%)
Sex							
Male	48,696 (50.9)	9,349 (51.0)	8,408 (50.2)	5,296 (50.8)	8,756 (50.7)	8,706 (52.2)	8,181 (50.9)
Female	46,890 (49.1)	9,023 (49.0)	8,147 (49.8)	5,149 (49.2)	8,500 (49.3)	8,114 (47.8)	7,957 (49.1)
Age (months) [b]	36.4 ± 13.5	36.3 ± 13.5	36.3 ± 13.6	36.8 ± 13.3	36.1 ± 13.4	36.4 ± 13.5	36.6 ± 13.6
Age group							
13 - <24	21,505 (22.3)	4,095 (22.7)	3,792 (22.9)	2,217 (20.7)	3,930 (23.1)	3,799 (22.1)	3,672 (22.2)
24 - <36	24,029 (25.2)	4,661 (25.1)	4,162 (25.2)	2,739 (26.3)	4,316 (24.6)	4,204 (25.4)	3,947 (24.7)
36 - <48	24,914 (26.1)	4,791 (26.3)	4,253 (25.6)	2,695 (25.8)	4,694 (27.0)	4,368 (26.1)	4,113 (25.8)
48 - <60	25,138 (26.4)	4,825 (25.9)	4,348 (26.4)	2,794 (27.2)	4,316 (25.4)	4,449 (26.4)	4,406 (27.3)
Natural region of residence							
Lima Metropolitana	11,931 (27.2)	2,315 (27.7)	2,107 (27.8)	1,431 (32.5)	2,163 (26.3)	2,018 (25.0)	1,897 (23.8)
Coast	28,471 (27.3)	5,472 (27.1)	5,041 (26.7)	3,164 (26.2)	5,070 (28.2)	4,906 (27.7)	4,818 (28.2)
Highlands	31,274 (27.5)	6,228 (28.1)	5,339 (27.6)	3,258 (24.7)	5,696 (27.6)	5,593 (28.8)	5,160 (27.9)
Jungle	23,910 (18.0)	4,357 (17.1)	4,068 (17.8)	2,592 (16.7)	4,327 (17.9)	4,303 (18.5)	4,263 (20.1)
Area of residence							
Urban	66,832 (74.0)	12,786 (73.8)	11,904 (73.5)	7,353 (76.2)	11,806 (74.2)	11,576 (73.7)	11,407 (73.1)
Rural	28,754 (26.0)	5,586 (26.2)	4,651 (26.6)	3,092 (23.8)	5,450 (25.9)	5,244 (26.3)	4,731 (26.9)
Family size (members)							
Small (<4)	18,087 (18.2)	3,109 (16.5)	3,052 (18.1)	1,767 (17.1)	3,359 (18.2)	3,425 (19.7)	3,375 (20.3)
Medium (4 - 5)	61,030 (63.3)	11,817 (63.8)	10,317 (61.7)	6,719 (63.5)	11,074 (63.9)	10,800 (63.9)	10,303 (63.1)
Large (≥6)	16,469 (18.5)	3,446 (19.8)	3,186 (20.3)	1,959 (19.5)	2,823 (17.9)	2,595 (16.4)	2,460 (16.6)
Mother's age at first delivery							
<18	21,815 (21.0)	4,202 (20.8)	3,775 (21.4)	2,317 (19.4)	3,887 (20.9)	3,931 (21.5)	3,703 (21.6)
18 - 30	68,155 (72.1)	13,072 (71.7)	11,801 (71.7)	7,549 (73.8)	12,343 (72.2)	11,944 (72.1)	11,446 (71.6)
>30	5,616 (6.9)	1,098 (7.6)	979 (7.0)	579 (6.8)	1,026 (7.0)	945 (6.3)	989 (6.9)

All proportions were weighted. [a] Some variables may add less than 95,586 due to missing data. [b] Mean ± standard deviation.

Table S3. Socioeconomic, health and access program of Peruvian children <5 years old between 2018 and 2023 (N=95,586) [a]

Characteristics	N=95,586 n (%)	DHS 2018 n=18,372 n (%)	2019 n=16,555 n (%)	2020 n=10,445 n (%)	2021 n=17,256 n (%)	2022 n=16,820 n (%)	2023 n=16,138 n (%)
Mother's educational							
Primary/no education	18,986 (19.0)	4,186 (21.4)	3,369 (20.6)	2,082 (17.3)	3,353 (18.6)	3,202 (18.3)	2,794 (17.0)
Secondary	45,093 (46.2)	8,188 (43.5)	7,692 (45.7)	4,947 (46.9)	8,165 (46.4)	8,222 (47.9)	7,879 (47.7)
Higher	31,507 (34.8)	5,998 (35.0)	5,494 (33.7)	3,416 (35.9)	5,738 (35.0)	5,396 (33.8)	5,465 (35.3)
Wealth index [b]	0.24 ± 0.99	0.26 ± 0.99	0.24 ± 0.98	0.33 ± 0.98	0.24 ± 0.99	0.19 ± 0.99	0.19 ± 0.99
Wealth index (quintile)							
Most poor	27,644 (24.9)	5,349 (24.9)	4,464 (24.9)	2,842 (21.2)	5,165 (25.0)	5,104 (26.5)	4,720 (26.8)
Poor	25,278 (23.8)	4,733 (23.1)	4,522 (24.7)	2,744 (23.4)	4,507 (23.2)	4,515 (24.4)	4,257 (24.1)
Middle	19,001 (20.2)	3,554 (19.7)	3,361 (20.0)	2,166 (21.2)	3,345 (20.6)	3,365 (20.5)	3,210 (19.6)
Rich	14,077 (17.2)	2,737 (17.3)	2,450 (16.6)	1,548 (17.9)	2,576 (17.6)	2,327 (16.4)	2,439 (17.2)
Most rich	9,586 (13.9)	1,999 (15.0)	1,758 (13.8)	1,145 (16.3)	1,663 (13.6)	1,509 (12.3)	1,512 (12.3)
Place of delivery							
At home	5,504 (6.1)	1,230 (7.1)	934 (6.5)	528 (4.8)	921 (5.7)	991 (6.0)	900 (5.9)
Health facility	88,753 (94.0)	16,930 (92.9)	15,356 (93.5)	9,784 (95.3)	16,071 (94.3)	15,578 (94.0)	15,034 (94.1)
Antenatal care (visits)							
0 - 6	14,718 (18.3)	2,621 (16.3)	2,301 (16.8)	1,341 (15.0)	2,684 (19.4)	3,078 (22.5)	2,693 (20.2)
7 - 12	57,833 (70.6)	10,982 (71.6)	10,030 (70.4)	6,627 (74.1)	10,373 (70.0)	9,925 (68.2)	9,896 (69.5)
≥13	8,278 (11.1)	1,665 (12.1)	1,627 (12.8)	895 (10.9)	1,452 (10.6)	1,318 (9.3)	1,321 (10.2)
Access to social programs							
No	58,183 (64.3)	11,097 (64.6)	10,373 (65.0)	7,135 (72.3)	10,308 (62.6)	9,838 (61.5)	9,432 (60.1)
Yes	37,403 (35.7)	7,275 (35.4)	6,182 (35.0)	3,310 (27.7)	6,948 (37.4)	6,982 (38.5)	6,706 (39.9)
Information access [c]							
No	10,160 (9.1)	1,486 (7.1)	1,390 (8.0)	976 (7.6)	1,804 (9.0)	2,106 (10.7)	2,398 (13.1)
Yes	85,426 (90.9)	16,886 (92.9)	15,165 (92.0)	9,469 (92.4)	15,452 (91.0)	14,714 (89.3)	13,740 (87.0)
Complete pneumococcal vaccinated							
No	28,175 (28.9)	5,583 (29.5)	4,621 (28.1)	3,218 (30.6)	5,212 (29.4)	4,942 (27.9)	4,599 (27.8)
Yes	67,411 (71.1)	12,789 (70.5)	11,934 (71.9)	7,227 (69.4)	12,044 (70.6)	11,878 (72.1)	11,539 (72.2)

All proportions were weighted. [a] Some variables may add less than 95,586 due to missing data. [b] Mean ± standard deviation. [c] Access to internet, TV and radio.

Table S4. Complete pneumococcal vaccination coverage in Peruvian children <5 years according to DHS period and region

Administrative region	Complete pneumococcal vaccination coverage					
	2018	2019	2020	2021	2022	2023
	%	%	%	%	%	%
Amazonas	69.4	74.8	70.9	70.4	67.9	64.5
Ancash	78.9	76.8	69.9	72.4	75.9	79.1
Apurimac	74.1	72.0	72.6	72.9	75.1	77.0
Arequipa	68.5	71.4	68.1	68.4	67.1	72.7
Ayacucho	71.6	78.2	69.9	73.9	72.0	73.3
Cajamarca	78.4	81.5	71.5	78.0	77.1	70.7
Callao	65.6	68.6	64.3	67.2	67.3	71.5
Cusco	76.8	77.3	75.2	75.3	74.2	72.5
Huancavelica	73.3	75.3	73.7	75.3	74.1	75.4
Huánuco	83.7	83.0	78.7	76.9	77.8	75.4
Ica	56.1	65.5	65.6	67.9	63.5	64.0
Junín	70.2	73.0	74.6	72.3	77.6	74.9
La Libertad	73.6	76.1	77.8	73.6	81.2	72.3
Lambayeque	66.0	67.7	67.5	66.5	69.9	68.9
Lima	67.6	69.4	66.3	70.1	72.2	73.8
Loreto	73.3	66.3	61.2	61.3	68.1	71.0
Madre de Dios	57.1	62.1	50.9	54.5	54.4	57.0
Moquegua	62.8	68.2	71.8	66.9	64.6	71.2
Pasco	63.1	72.5	63.2	69.2	63.7	68.1
Piura	78.3	74.9	78.2	76.5	77.3	77.2
Puno	57.5	54.9	58.0	58.6	54.7	53.3
San Martin	77.6	81.8	78.9	74.4	73.1	78.7
Tacna	64.4	67.6	65.9	66.3	69.3	73.4
Tumbes	71.1	76.5	73.8	75.1	77.8	77.7
Ucayali	61.1	65.5	61.4	61.7	61.9	62.9

Table S5. Demographic and sociodemographic characteristics associated with complete pneumococcal vaccination coverage in Peruvian children <5 years old between 2018 and 2023 (N=95,586)

Characteristics	Bivariate analysis			Multivariate analysis [a]			Interaction analysis [b]		
	PR	CI 95%	p	aPR	CI 95%	p	PR	CI 95%	p
Sex									
Male	Ref.			Ref.					
Female	1.00	0.99 - 1.01	0.815	1.01	0.99 - 1.02	0.327			
Age (months)	1.00	1.00 - 1.00	<0.001	-	-	-			
Age group (months)									
13 - <24	Ref.			Ref.					
24 - <36	1.02	1.01 - 1.04	0.003	1.02	1.00 - 1.03	0.025			
36 - <48	0.97	0.96 - 0.99	<0.001	0.97	0.96 - 0.99	<0.001			
48 - <60	0.93	0.92 - 0.95	<0.001	0.93	0.91 - 0.94	<0.001			
Natural region of residence									
Lima Metropolitana	Ref.			Ref.			Ref.		
Coast	1.04	1.02 - 1.06	<0.001	1.04	1.02 - 1.06	<0.001	0.99	0.96 - 1.01	0.346
Highlands	1.05	1.03 - 1.07	<0.001	1.00	0.98 - 1.02	0.827	0.96	0.94 - 0.99	0.008
Jungle	1.03	1.01 - 1.05	0.015	1.06	1.04 - 1.08	<0.001	1.02	0.99 - 1.06	0.052
Type of residence									
Urban	Ref.			Ref.					
Rural	1.03	1.01 - 1.05	<0.001	1.04	1.02 - 1.06	<0.001			
Family size (members)									
Small (<4)	Ref.			Ref.					
Medium (4 - 5)	0.94	0.93 - 0.96	<0.001	0.95	0.93 - 0.96	<0.001			
Large (≥6)	0.84	0.82 - 0.86	<0.001	0.87	0.84 - 0.88	<0.001			
Mother's age at first delivery									
<18	Ref.			Ref.					
18 - 30	1.11	1.10 - 1.13	<0.001	1.08	1.07 - 1.10	<0.001			
>30	1.20	1.17 - 1.22	<0.001	1.15	1.12 - 1.18	<0.001			
Mother's educational									
Primary/no education	Ref.			Ref.					
Secondary	1.00	0.99 - 1.02	0.639	0.98	0.96 - 0.99	0.006			
Higher	1.04	1.02 - 1.06	<0.001	0.99	0.97 - 1.01	0.362			
Wealth index	1.01	1.00 - 1.02	0.024	-	-	-			
Wealth index (quintile)									
Most poor	1.00	0.98 - 1.02	0.876	0.97	0.94 - 0.99	0.002			
Poor	1.00	0.99 - 1.02	0.660	0.98	0.96 - 0.99	0.009			
Middle	Ref.			Ref.					
Rich	1.01	0.99 - 1.03	0.224	1.01	0.99 - 1.03	0.216			
Most rich	1.01	0.98 - 1.03	0.575	1.01	0.98 - 1.03	0.608			
Place of delivery									
At home	Ref.			Ref.					
Health facility	1.17	1.12 - 1.21	<0.001	1.11	1.07 - 1.14	<0.001			
Antenatal care (visits)									
0 - 6	Ref.			Ref.					
7 - 12	1.14	1.12 - 1.16	<0.001	1.12	1.10 - 1.14	<0.001			
≥13	1.17	1.15 - 1.20	<0.001	1.15	1.12 - 1.17	<0.001			
Access to social programs									
No	Ref.			Ref.					
Yes	1.13	1.11 - 1.14	<0.001	1.15	1.14 - 1.17	<0.001			
Information access									
No	Ref.			Ref.					
Yes	1.10	1.08 - 1.13	<0.001	1.09	1.06 - 1.11	<0.001			
DHS period									
2018	1.02	0.99 - 1.04	0.184	1.00	0.98 - 1.02	0.971			
2019	1.04	1.01 - 1.06	0.003	1.04	1.01 - 1.06	0.004			
2020	Ref.			Ref.					
2021	1.02	0.99 - 1.04	0.158	1.01	0.98 - 1.03	0.508			
2022	1.04	1.02 - 1.06	0.001	1.03	1.01 - 1.06	0.006			
2023	1.04	1.02 - 1.07	0.001	1.03	1.00 - 1.05	0.026			

PR: Prevalence ratio. aPR: Adjusted Prevalence ratio. 95% CI: 95% confidence interval. Ref: Reference category. Bivariate and multivariate analysis using linearized variance (modified) Poisson regression.

[a] Adjusted by sex, age group, natural region of residence, type of residence, family size, mother's age at first delivery, mother's educational, wealth index, place of delivery, antenatal care, access to social programs, information access and DHS period.

[b] The interaction analysis was performed based on the multivariate model to assess whether the effect of wealth (continuous) on CPVC varied by natural region.

Table S6. Erreygers normalized concentration index for complete pneumococcal vaccination coverage according to wealth index (quintiles) between 2018 and 2023

Administrative region	Erreygers normalized concentration index																	
	DHS-2018			DHS-2019			DHS-2020			DHS-2021			DHS-2022			DHS-2023		
	ECi	CI 95%	p	ECi	CI 95%	p	ECi	CI 95%	p	ECi	CI 95%	p	ECi	CI 95%	p	ECi	CI 95%	p
Nationwide	-0.026	-0.051 - -0.002	0.036	-0.007	-0.032 - 0.018	0.574	-0.015	-0.047 - 0.016	0.340	0.040	0.015 - 0.065	0.002	0.035	0.012 - 0.059	<0.001	0.048	0.023 - 0.072	<0.001
Amazonas	0.222	0.111 - 0.333	<0.001	0.165	0.057 - 0.272	0.003	0.219	0.045 - 0.393	0.014	0.187	0.065 - 0.309	0.003	0.340	0.210 - 0.470	<0.001	0.443	0.335 - 0.551	<0.001
Ancash	-0.037	-0.119 - 0.045	0.377	0.015	-0.060 - 0.090	0.694	-0.100	-0.242 - 0.042	0.166	-0.052	-0.161 - 0.056	0.341	-0.091	-0.190 - 0.009	0.074	-0.096	-0.182 - -0.010	0.030
Apurimac	0.036	-0.060 - 0.132	0.462	0.035	-0.063 - 0.133	0.482	-0.023	-0.126 - 0.080	0.659	0.042	-0.057 - 0.141	0.401	0.009	-0.079 - 0.096	0.846	0.001	-0.092 - 0.094	0.985
Arequipa	0.073	-0.016 - 0.161	0.105	0.096	0.013 - 0.180	0.024	0.111	-0.046 - 0.268	0.162	0.068	-0.041 - 0.177	0.217	0.061	-0.048 - 0.169	0.272	0.022	-0.054 - 0.097	0.566
Ayacucho	0.044	-0.051 - 0.139	0.360	-0.116	-0.209 - -0.024	0.014	0.063	-0.030 - 0.156	0.182	0.044	-0.040 - 0.127	0.305	0.012	-0.066 - 0.091	0.757	0.045	-0.048 - 0.137	0.341
Cajamarca	0.061	-0.028 - 0.150	0.175	0.035	-0.068 - 0.138	0.502	0.081	-0.059 - 0.219	0.252	0.035	-0.059 - 0.128	0.465	-0.029	-0.123 - 0.065	0.544	0.111	0.014 - 0.208	0.025
Callao	0.039	-0.046 - 0.124	0.362	-0.016	-0.109 - 0.077	0.735	0.154	0.032 - 0.275	0.014	0.027	-0.071 - 0.125	0.588	0.094	0.007 - 0.182	0.035	0.026	-0.054 - 0.106	0.515
Cusco	-0.038	-0.123 - 0.046	0.372	-0.047	-0.132 - 0.038	0.272	-0.055	-0.202 - 0.092	0.457	-0.008	-0.091 - 0.075	0.846	0.025	-0.066 - 0.116	0.591	-0.040	-0.144 - 0.063	0.442
Huancavelica	-0.028	-0.125 - 0.069	0.565	-0.011	-0.121 - 0.100	0.851	0.103	-0.014 - 0.221	0.084	0.019	-0.072 - 0.109	0.681	0.053	-0.042 - 0.148	0.272	0.038	-0.068 - 0.144	0.482
Huánuco	-0.002	-0.070 - 0.065	0.946	-0.032	-0.093 - 0.030	0.308	0.076	-0.058 - 0.211	0.263	0.023	-0.066 - 0.112	0.604	0.036	-0.061 - 0.132	0.464	0.026	-0.056 - 0.107	0.531
Ica	0.059	-0.044 - 0.162	0.261	0.200	0.116 - 0.284	<0.001	-0.010	-0.127 - 0.107	0.864	-0.004	-0.089 - 0.081	0.923	0.114	0.003 - 0.224	0.043	0.023	-0.077 - 0.123	0.654
Junín	0.039	-0.066 - 0.144	0.461	0.078	-0.038 - 0.194	0.185	0.067	-0.063 - 0.197	0.307	-0.017	-0.102 - 0.069	0.700	0.014	-0.067 - 0.095	0.736	0.051	-0.031 - 0.133	0.222
La Libertad	-0.018	-0.131 - 0.096	0.760	0.000	-0.095 - 0.096	0.997	0.048	-0.059 - 0.155	0.376	0.122	0.019 - 0.225	0.021	0.028	-0.077 - 0.133	0.599	0.035	-0.073 - 0.142	0.528
Lambayeque	0.069	-0.054 - 0.192	0.268	0.053	-0.052 - 0.157	0.320	0.048	-0.066 - 0.162	0.406	-0.027	-0.134 - 0.081	0.623	0.031	-0.091 - 0.153	0.617	0.097	-0.064 - 0.258	0.237
Lima	0.039	-0.014 - 0.092	0.147	0.052	-0.008 - 0.111	0.088	-0.023	-0.085 - 0.040	0.474	0.080	0.023 - 0.136	0.006	0.086	0.039 - 0.133	<0.001	0.032	-0.020 - 0.085	0.223
Loreto	0.028	-0.076 - 0.132	0.597	0.073	-0.036 - 0.182	0.185	0.113	-0.053 - 0.280	0.179	0.222	0.090 - 0.355	0.001	0.230	0.115 - 0.346	<0.001	0.270	0.160 - 0.380	<0.001
Madre de Dios	0.100	0.002 - 0.197	0.045	0.012	-0.082 - 0.107	0.795	0.087	-0.061 - 0.236	0.245	0.059	-0.048 - 0.167	0.277	0.080	-0.045 - 0.204	0.207	0.145	0.033 - 0.257	0.012
Moquegua	0.031	-0.069 - 0.131	0.543	-0.038	-0.122 - 0.047	0.378	0.008	-0.119 - 0.135	0.901	0.087	-0.004 - 0.178	0.061	0.035	-0.077 - 0.147	0.536	-0.036	-0.126 - 0.054	0.425
Pasco	-0.059	-0.174 - 0.057	0.317	-0.029	-0.129 - 0.071	0.568	0.028	-0.114 - 0.169	0.700	0.027	-0.068 - 0.122	0.574	-0.015	-0.133 - 0.102	0.799	0.015	-0.089 - 0.119	0.775
Piura	-0.039	-0.122 - 0.043	0.348	-0.116	-0.208 - -0.025	0.013	-0.049	-0.150 - 0.052	0.338	0.059	-0.025 - 0.144	0.168	-0.063	-0.168 - 0.043	0.242	-0.030	-0.127 - 0.067	0.544
Puno	-0.190	-0.311 - -0.069	0.002	-0.087	-0.212 - 0.039	0.176	-0.197	-0.343 - -0.051	0.009	-0.113	-0.225 - -0.001	0.048	-0.045	-0.163 - 0.074	0.456	0.001	-0.116 - 0.117	0.989
San Martin	0.021	-0.078 - 0.120	0.674	0.082	-0.017 - 0.181	0.103	0.056	-0.038 - 0.150	0.239	0.073	-0.030 - 0.177	0.161	0.139	0.033 - 0.244	0.010	0.067	-0.028 - 0.162	0.164
Tacna	0.038	-0.099 - 0.175	0.584	0.024	-0.076 - 0.124	0.639	0.065	-0.096 - 0.226	0.422	0.085	0.004 - 0.167	0.040	0.102	0.012 - 0.193	0.028	-0.035	-0.123 - 0.053	0.434
Tumbes	0.028	-0.053 - 0.109	0.498	0.112	0.020 - 0.204	0.017	-0.022	-0.142 - 0.098	0.714	-0.003	-0.087 - 0.081	0.943	0.017	-0.060 - 0.095	0.655	0.002	-0.082 - 0.086	0.963
Ucayali	0.232	0.152 - 0.312	<0.001	0.192	0.104 - 0.281	<0.001	0.219	0.113 - 0.325	<0.001	0.367	0.264 - 0.470	<0.001	0.279	0.181 - 0.378	<0.001	0.228	0.142 - 0.314	<0.001

ECi: Erreygers normalized concentration index.
p<0.05: Index probability is different from zero (inequality).

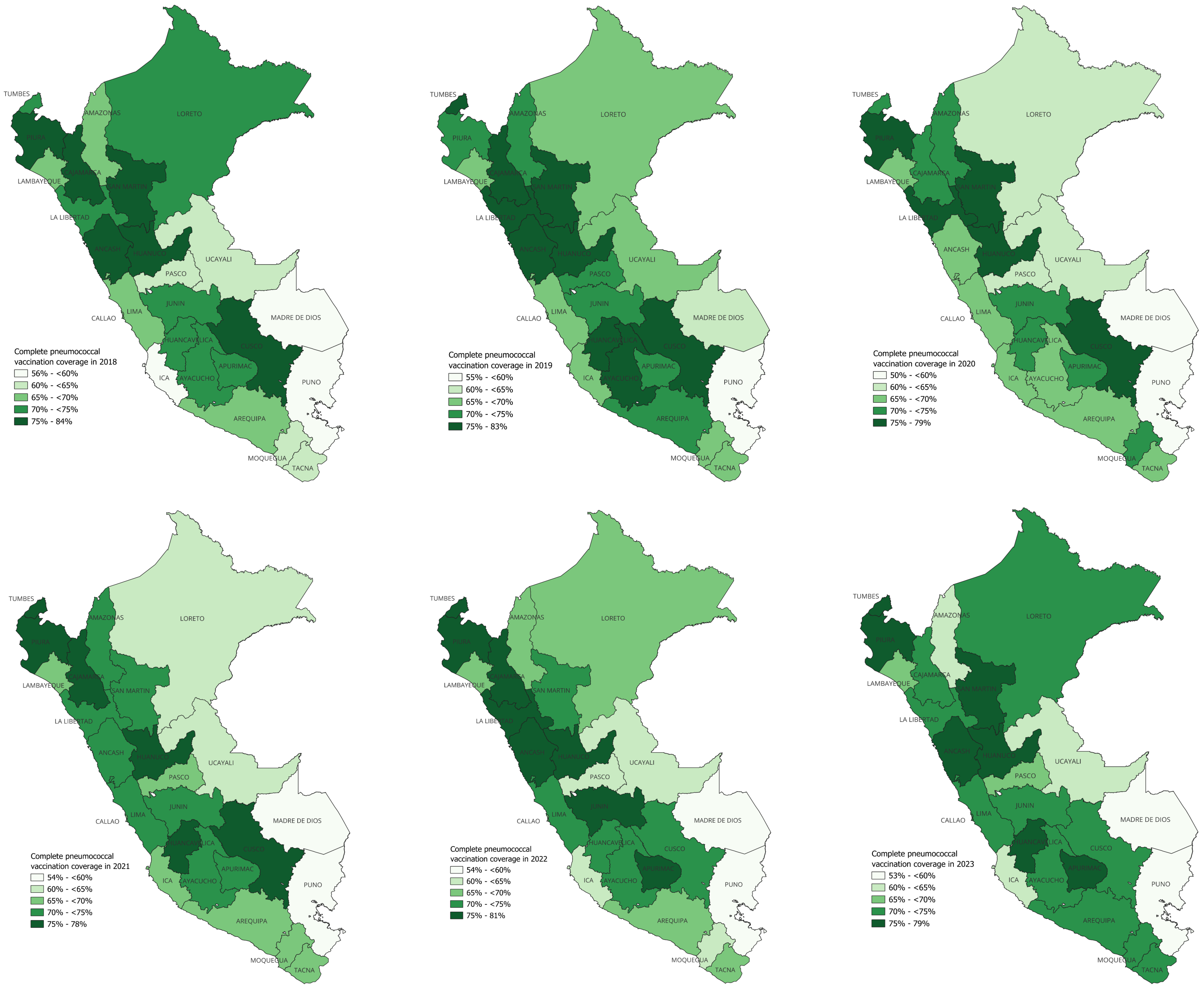

Figure S1. Complete pneumococcal vaccination coverage in Peruvian children <5 years old between 2018 and 2023 by region

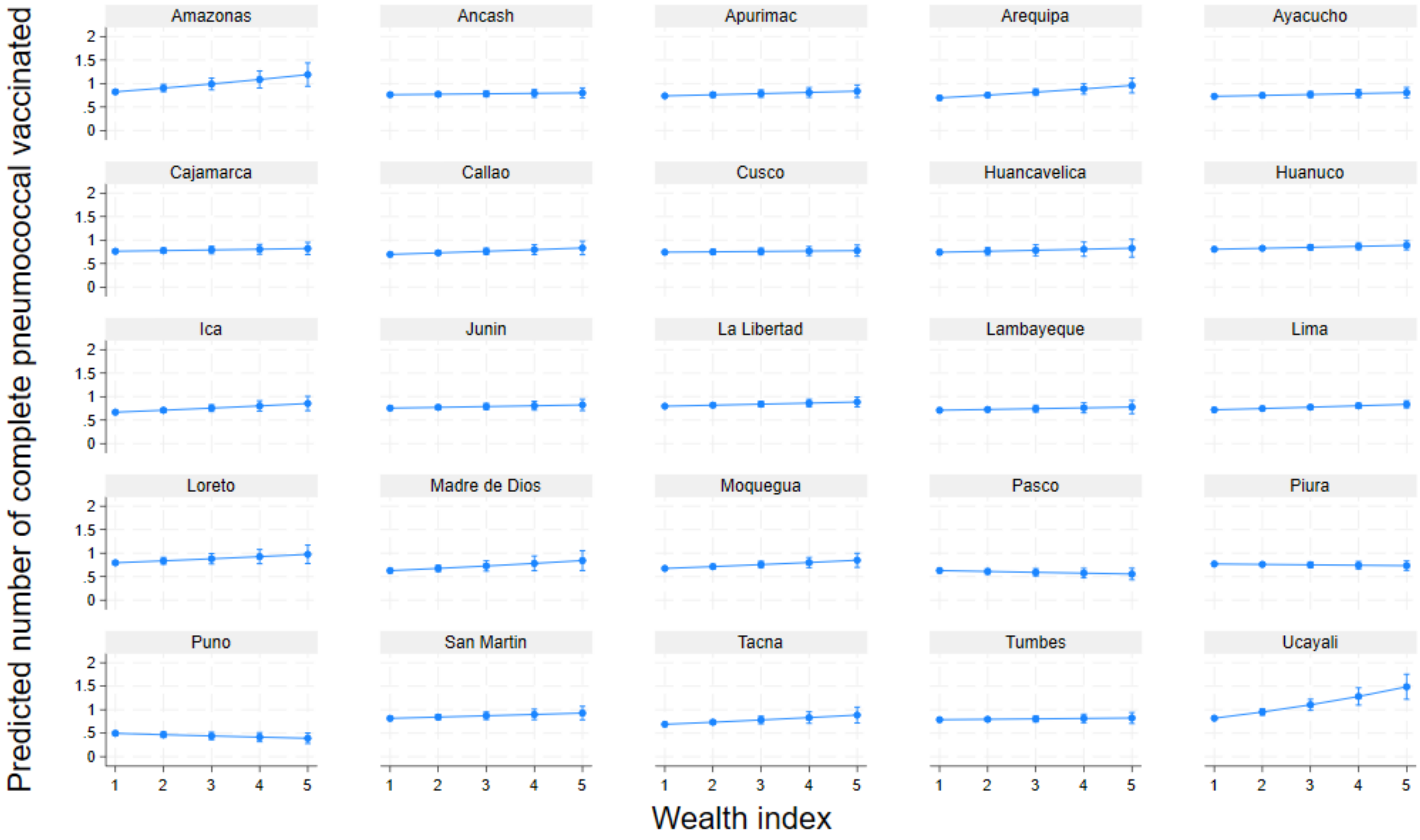

Figure S2. Predictive margins plot of interaction effects of wealth on complete pneumococcal vaccination coverage varied by administrative region with 95% confidence interval
